# Protective effect of resveratrol on mitochondrial biogenesis during hyperoxia-induced brain injury in neonatal pups

**DOI:** 10.1186/s12868-023-00797-1

**Published:** 2023-04-25

**Authors:** Menghan Yang, Yunchuan Shen, Shuai Zhao, Rong Zhang, Wenbin Dong, Xiaoping Lei

**Affiliations:** 1 Division of Neonatology, Department of Pediatrics, The Affiliated Hospital of Southwest Medical University, No. 8, Section 2, Kangcheng Road, Luzhou, Sichuan 646000 China; 2 Department of Perinatology, The Affiliated Hospital of Southwest Medical University, Luzhou, Sichuan China; 3 Sichuan Clinical Research Center for Birth Defects, Luzhou, Sichuan China

**Keywords:** Hyperoxia, Brain, Neonatal rats, Resveratrol, PGC-1α, Mitochondrial biogenesis

## Abstract

**Background:**

Neonatal hyperoxic brain injury is caused by exposure to hyperphysiological oxygen content during the period of incomplete development of the oxidative stress defence system, resulting in a large number of reactive oxygen species (ROS) and causing damage to brain tissue. Mitochondrial biogenesis refers to the synthesis of new mitochondria from existing mitochondria, mostly through the PGC-1α/Nrfs/TFAM signalling pathway. Resveratrol (Res), a silencing information regulator 2-related enzyme 1 (Sirt1) agonist, has been shown to upregulate the level of Sirt1 and the expression of peroxisome proliferator-activated receptor gamma coactivator-1α (PGC-1α). We speculate that Res has a protective effect on hyperoxia-induced brain injury through mitochondrial biogenesis.

**Methods:**

Sprague-Dawley (SD) pups were randomly divided into the nonhyperoxia (NN) group, the nonhyperoxia with dimethyl sulfoxide (ND) group, the nonhyperoxia with Res (NR) group, the hyperoxia (HN) group, the hyperoxia with dimethyl sulfoxide (HD) group, and the hyperoxia with Res (HR) group within 12 h after birth. The HN, HD, and HR groups were placed in a high-oxygen environment (80‒85%), and the other three groups were placed in the standard atmosphere. The NR and HR groups were given 60 mg/kg Res every day, the ND and HD groups were given the same dose of dimethyl sulfoxide (DMSO) every day, and the NN and HN groups were given the same dose of normal saline every day. On postnatal day (PN) 1, PN7, and PN14, brain samples were acquired for HE staining to assess pathology, TUNEL to detect apoptosis, and real-time quantitative polymerase chain reaction and immunoblotting to detect the expression levels of Sirt1, PGC-1α, nuclear respiratory factor 1 (Nrf1), nuclear respiratory factor 2 (Nrf2) and mitochondrial transcription factor A (TFAM) in brain tissue.

**Results:**

Hyperoxia induced brain tissue injury; increased brain tissue apoptosis; inhibited Sirt1, PGC-1α, Nrf1, Nrf2, TFAM mRNA expression in mitochondria; diminished the ND1 copy number and ND4/ND1 ratio; and decreased Sirt1, PGC-1α, Nrf1, Nrf2, and TFAM protein levels in the brain. In contrast, Res reduced brain injury and attenuated brain tissue apoptosis in neonatal pups and increased the levels of the corresponding indices.

**Conclusion:**

Res has a protective effect on hyperoxia-induced brain injury in neonatal SD pups by upregulating Sirt1 and stimulating the PGC-1α/Nrfs/TFAM signalling pathway for mitochondrial biogenesis.

**Supplementary Information:**

The online version contains supplementary material available at 10.1186/s12868-023-00797-1.

## Introduction

Neonatal hyperoxic organ injury is caused by oxidative stress damage to systemic organs through excessive exposure to oxygen during the neonatal period. At present, studies have confirmed that hyperoxia can cause damage to the lungs, brain, kidneys, eyes, intestines, and other organs, especially in premature infants. An animal experiment using a hyperoxia model of neonatal mice confirmed that hyperoxia can lead to thymus damage, affect T-cell development and lead to adaptive immune system hypoplasia. [1] The prevention of and protection against hyperoxia-induced organ injury are particularly significant.

Among these organ injuries, brain injury has a great impact on newborns, and neurodevelopmental damage will accompany them for a lifetime. In recent years, studies have shown that oxidative stress caused by hyperoxia can affect various brain tissues in many ways. Hyperoxia can lead to the activation of nod-like receptor pyrin domain-containing-3 (NLRP3) inflammatory bodies in brain tissue, resulting in neuronal damage and cell death. [2] The increase in oxygen partial pressure in a hyperoxic environment will cause delayed development of white matter and impaired axonal conduction in mice [3] In addition, hyperoxia can damage the cells in the hippocampus, destroy the development of hippocampal neurons, affect the balance of excitatory and inhibitory nerve transmission in the hippocampus, and produce learning and cognitive impairment. [4] The cerebral vascular structure and function of mice with hyperoxia-induced experimental bronchopulmonary dysplasia (BPD) showed lifelong damage, with a long-term decline in motor and cognitive function. [5].

Oxidative stress attacks on newborns can be divided into two types, oxidative stress caused by the transformation of the intrauterine and extrauterine environment during delivery and oxidative stress caused by hyperoxia therapy. A large number of studies have confirmed that the damage source of oxidative stress damage, ROS, is a byproduct of incomplete reduction of mitochondrial respiratory chain complexes I and III in the process of molecular oxygen receiving electrons and being reduced to water. [6, 7] A large amount of ROS can destroy the structure and function of important molecules, such as mitochondrial DNA (mtDNA), the plasma membrane, and the respiratory chain complex in mitochondria, and cause cells to be in a state of oxidative stress. [8] Therefore, maintaining mitochondrial homeostasis plays a key role in reducing oxidative stress damage.

Mitochondrial homeostasis is also known as mitochondrial quality control, which includes mitochondrial biogenesis, mitochondrial fusion division, and mitochondrial autophagy. Mitochondrial biogenesis is the process of synthesizing new mitochondria from existing mitochondria. By adjusting the expression of the pathway, the number of mitochondria is changed according to the needs of cells to maintain cell balance. The key factor in mitochondrial biogenesis is PGC-1α, which is expressed in tissues with high energy requirements. Studies have shown that in different cancer cells, the upregulation of PGC-1α can protect cells from the production of too much ROS and promote cell survival. [9] Mice lacking PGC-1α were more sensitive to oxidative damage. [10].

The activation of downstream Nrf1, Nrf2 and TFAM improves mtDNA replication and completes mitochondrial biogenesis. The PGC-1α/Nrf/TFAM/mtDNA signalling pathway has been confirmed in many disease models, such as peritoneal fibrosis associated with peritoneal dialysis, type 2 diabetes mellitus, and ulcerative colitis. [11–13].

At present, a number of targets have been confirmed to activate PGC-1α in the brain, including brain-derived neurotrophic factor (BDNF), insulin receptor substrate (IRS), oxalyl acetate (OAA), thyroid hormone (TH), melatonin, and Sirt1. [14–19] Among them, Sirt1 is the classical upstream molecule of PGC-1α. Sirt1 exists mainly in the nuclei of most cell types and is a member of the sirtuin protein family. It activates the expression of PGC-1α by deacetylating lysine residues. [20] Res is a stilbene natural polyphenol and one of the natural agonists of Sirt1. It has a variety of therapeutic effects, including anti-inflammation, antioxidation, antiplatelet, antihyperlipidaemia, immunomodulatory, anti-carcinogenicity, cardioprotective, vascular relaxant, and neuroprotective effects. [21] Because of its ability to cross the blood-brain barrier and play a role in neurons and glial cells, Res and its related polyphenols have been studied in a variety of nervous system disease models. For example, Res can regulate ROS and neurotransmitters in the hypothalamic paraventricular nucleus to reduce sympathetic activity and blood pressure, [22] inhibit the electrophysiological activity of acid ion channels in pain neurons to reduce peripheral pain, [23] and reverse the learning and memory impairment of neonatal rats caused by the inhaled anaesthetic sevoflurane. [24] Res and its derivatives have fewer clinical applications in nervous system diseases and more applications in mild coronaviruses, such as COVID-19; type 2 diabetes nonalcoholic fatty liver disease; and other diseases. [25–27].

Our group has previously confirmed that Res has a protective effect on hyperoxia-induced brain injury in neonatal rats, but the pathway through which it acts has not been confirmed. [28, 29] Therefore, on the basis of previous research progress, we verified the changes in Sirt1, PGC-1α, Nrf1, Nrf2, and TFAM in the brain tissue of neonatal pups in a hyperoxia injury model and clarified that Res can activate the mitochondrial biogenetic pathway through the Sirt1 site, which has a protective effect on hyperoxia-induced brain injury in neonatal pups.

## Materials and methods

### Animals and model establishment

The animals used for the following procedures were approved by the Laboratory Animal Ethics Committee of Southwestern Medical University, and SD rats were purchased from the Southwestern Medical University Laboratory Animal Center. Pregnant SD rats were housed individually in clear cages in the laboratory during the week prior to delivery, and pups were delivered spontaneously at a gestational age of 21 to 23 days. All pups were randomly divided into 6 groups with 27 pups in each group within 12 h after birth.

Referring to our group’s previous modelling method, [30, 31] the HN, HD, and HR groups were placed in a closed oxygen tank, and oxygen was introduced at a flow rate of 1–2 L/min to maintain an oxygen concentration of 85%. The environmental conditions in these groups were the same as those in the NN, ND, and NR groups, except for the inhalation of room air. Using alkaline lime to absorb CO_2_, the concentration of CO_2_ was kept below 0.5%, and the room temperature (25–27 °C), humidity (60–70%), and daily light and dark cycles were automatically monitored and adjusted. Mothers exposed to hyperoxia were exchanged with those exposed to nonhyperoxia every 24 h to avoid hyperoxia toxicity and to equalize their differences in care capacity and nutritional status. The dam, food, and drinking water were renewed every 24 h.

DMSO (Solarbio, Beijing) and saline were mixed in a 35:65 ratio to create a 35% DMSO solution, and Res (McLean, Shanghai) was prepared with 35% DMSO. 60 mg/Kg was injected intraperitoneally into the NR group and the HR group according to weight. The ND group and the HD group were injected with 35% DMSO every day, and the NN group and the HN group were injected with saline every day using the same dose, mode of administration, and time of administration as in the Res groups. The body weight and status of the pups in each group were observed and recorded every day.

### Sample preparation

On PN1, PN7, and PN14, three pups in each group were randomly selected for isoflurane anaesthesia (Raywald, China). Brain tissues were removed, frozen in liquid nitrogen and stored at -80℃. Another 6 pups were anaesthetized and exposed and inserted into the apex of the heart using a 0.4-gauge scalp needle into the aortic arch, and the right atrial appendage was cut and rinsed with saline, followed by perfusion with 4% paraformaldehyde for fixation. The brain tissues were removed and fixed overnight in paraformaldehyde (Biyuntian, Shanghai) before preparing paraffin sections and frozen sections.

### Histopathology

Brain tissues fixed with paraformaldehyde overnight were washed with water, dehydrated in an ethanol gradient and embedded in paraffin. Microtomes (Leica Biosystems, Germany) were used to prepare 4 μm thick tissue sections, which were dried for preservation. The sections were dewaxed and stained with haematoxylin and eosin (Solarbio, Beijing) and observed under a KF-PRO-002 digital slice scanner (Jiangfeng, China).

### Terminal-deoxynucleotidyl transferase-mediated nick end labelling (TUNEL) staining

Brain tissue fixed with paraformaldehyde overnight was washed with PBS, dehydrated with sucrose (BioFroxx, Germany) and OCT (SAKURA, Japan) in a gradient, and then frozen at -80 °C. Frozen sections with a thickness of 8 μm were prepared by a Leica CM1950 cryomicrotome (Leica Biosystems, Germany) and stored at -20 °C. Triton X-100 (BioFroxx, Germany) and protease K (Novozen, Nanjing) were used to permeate the cells, which were incubated with FITC-12-dUTP (Novozen, Nanjing) at 37 °C for 1 h for staining. DAPI (Biyuntian, Shanghai) was used to stain the cells in dark conditions, and glycerol (Solarbio, Beijing) was used to seal the slides. Under an inverted phase contrast fluorescence microscope (Olympus, Japan), 460 nm blue fluorescence and 520 nm green fluorescence were used for photo observation. The number of TUNEL-positive cells was calculated by ImageJ, and the apoptosis index was expressed as the number of apoptotic cells in a visual field/the total number of cells in the visual field.

### RNA isolation and real-time quantitative polymerase chain reaction (q-PCR)

RNA was extracted from fresh brain tissue by an RNA Easy Fast animal tissue/cell total RNA extraction kit (Tiangen, Beijing), and then the isolated RNA was subjected to RT-PCR using a HiScript® III RT SuperMix for qPCR kit (Novozem, Nanjing) to generate cDNA. The ChamQ Universal SYBR qPCR Master Mix Kit (Novozen, Nanjing) was used to react on a QuantStudioTM 3 Real-Time PCR instrument (Thermo, USA). The q-PCR cycle included predenaturation at 95 °C for 30 s; cycle reaction at 95 °C for 10 s and 60 °C for 30 s for 40 cycles; and melting at 95 °C for 15 s, 60 °C for 60 s, and 95 °C for 15 s. The primer sequences are shown in Table 1. The circulation thresholds of Sirt1, PGC-1α, Nrf1, Nrf2, TFAM, ND1, and ND4 were normalized according to the circulation threshold of GAPDH. The relative expression of the target gene was detected by 2^−ΔΔCT^, and the air group was used as the control group for data analysis.

**Table 1:** Primer sequences for q-PCR

Genes	Forward Primer(5’ → 3’)	Reverse Primer(5’ → 3’)
GAPDH	GAAGGTCGGTGTGAACGGAT	CCCATTTGATGTTAGCGGGAT
Sirt1	ATGGTATTTATGCTCGCCTTGC	GCTGAGTTGCTGGATTTTGTGT
PGC-1α	GAGGGACGAATACCGCAGAG	CTCTCAGTTCTGTCCGCGTT
Nrf1	GAGTGACCCAAACCGAACAC	TGCCGTGGAGTTGAGTATGT
Nrf2	CTTCATCTGGCCGCACAGTA	TCCACTTTGGTCCTGGCATC
TFAM	GTTGTCATTGGGATTGGGCA	CAAACGGCAGAACTCGTCAT
ND1	GCAGGACCATTCGCCCTATT	AAAACGGGGGTAGGATGCTC
ND4	AACCTAGCACTACCACCCCT	TCTCGTGTGTGGGAAGGTTG

### Western blot analysis

Total protein in fresh brain tissue was extracted by a total protein extraction kit (Solarbio, Beijing) and determined by a BCA protein quantitative kit (Solarbio, Beijing). After adjustment to the same concentration, protein samples were separated by electrophoresis on a 10% SDS-PAGE gel and transferred to an Immobilon-PSQ PVDF Transfer Membrane (Millipore, USA). Under the action of UltraSignal high-sensitivity ECL chemiluminescence substrate (Sizhengbai, Beijing), the protein was visualized by a chemiluminescence imager (VIBER LOURMAT, France). The antibodies used were as follows: GAPDH (1:10000 blue sky, Shanghai), Sirt1 (1:2000 Abcam, USA), PGC-1α (1:2000 Abcam, USA), Nrf1 (1:3000 CST, USA), and Nrf2 (1:2000 Abcam). ImageJ was used to measure the grey values of Sirt1, PGC-1α, Nrf1, Nrf2, and TFAM, which were normalized to the grey value of GAPDH. The data of the NN group were analysed as the control group.

### Statistical analysis

ImageJ was used to process data, SPSS 26.0 was used for statistical analysis, and GraphPad Prism 9.0 was used for drawing. All the experimental data are expressed as the mean and standard deviation. Data conforming to a normal distribution were tested by ANOVA and LSD, and data conforming to a skewed distribution were tested by the nonparametric Kruskal-Wallis test. *P* < 0.05 indicates statistical significance.

## Results

### Differences in body weight and status of young rats among groups

**Fig. 1:**
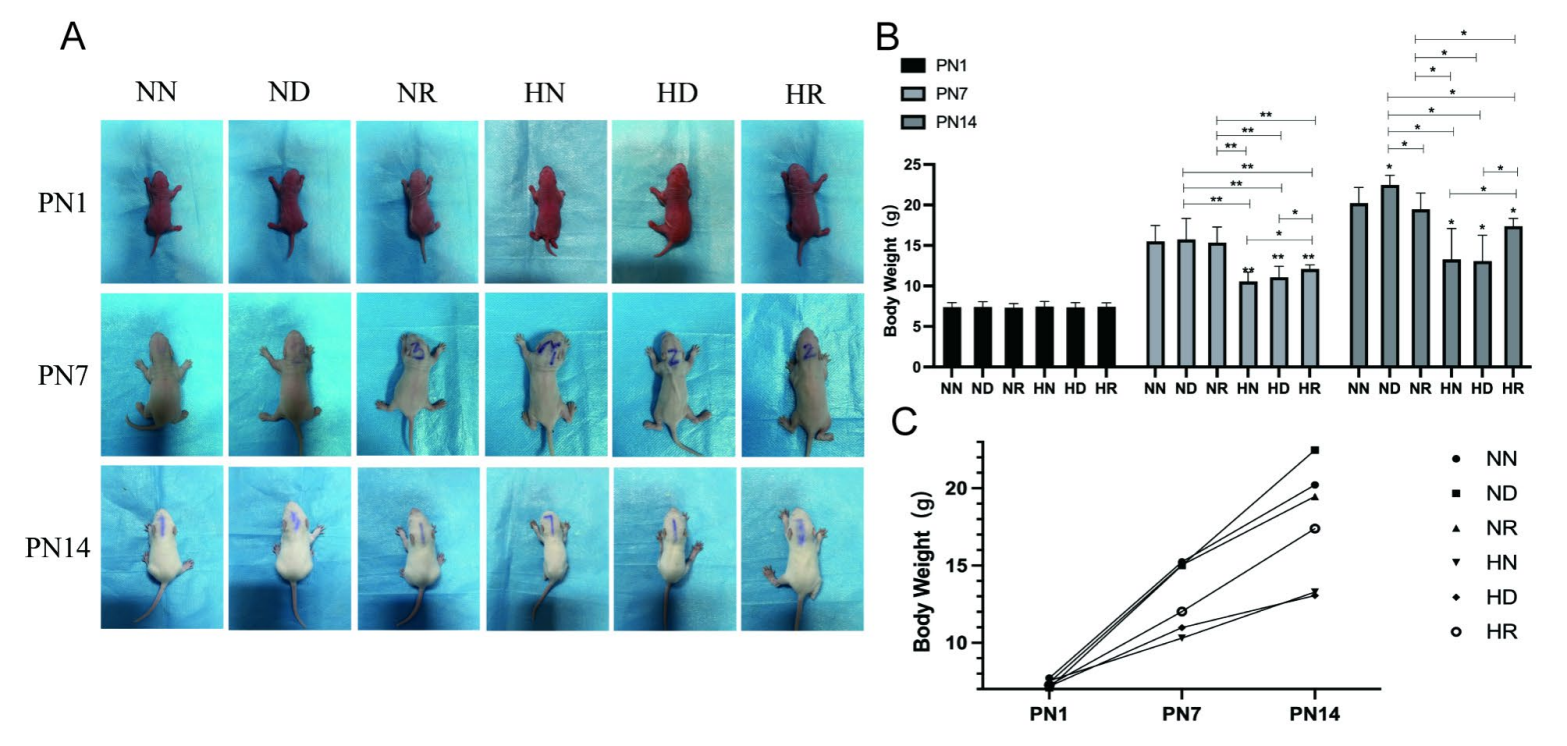
(**A**) photographs of SD pups. (**B**) weight bar chart of pups in NN, ND, NR, HN, HD, and HR groups on PN1, PN7, and PN14. *p < 0.05, **p < 0.001. (**C**) weight line chart of pups in NN, ND, NR, HN, HD, and HR groups on PN1, PN7, and PN14. NN, the nonhyperoxia group; ND, the nonhyperoxia with dimethyl sulfoxide group; NR, the nonhyperoxia with Res group; HN, the hyperoxia group; HD, the hyperoxia with dimethyl sulfoxide group; HR, the hyperoxia with Res group; PN1, postnatal day 1; PN7, postnatal day 7; PN14, postnatal day 14; Res, resveratrol.

Through the daily recording of the body weight and status of the pups, it was found that on PN1, the skin of the pups in each group was ruddy, and there was no significant difference in their states. At PN7, the body shape of the pups in the three groups in the hyperoxia environment was slightly smaller than that of the pups in the three groups in the air environment, and their movement was slow. On PN14, the pups in the HN group and the HD group were significantly smaller than those in the three groups in the air environment, with dull fur, wrinkled skin, low body temperature, cyanosis of the lips and limbs, head tremor and stumbling gait when placed on the plane. There was no significant difference in states between the pups in the HR group and those in the three groups in the air environment; they could crawl normally, and their bodies were slightly thinner (Fig. 1A). In the process of modelling, the mortality rate of pups in the HN group and the HD group was high. After removing the brains, the brains of the pups in these two groups were found to be smaller than those of the pups in the other four groups. The body weight analysis showed that there was no significant difference in body weight among the pups in all groups on PN1. On PN7, the body weights of the pups in the three groups in the hyperoxia environment were less than those of the pups in the air environment, and the data were significantly different, but there was no difference among the three groups in the shared environment. On PN14, the body weights of the pups in HN and HD groups were lower than those of the pups in the NN, ND, NR, and HR groups. The difference was statistically significant *(p < 0.05)*. There was no significant difference in body weight between the pups in the HR group and the pups in the three air groups. In the hyperoxic environment, the weight gain of the pups in the HN and HD groups was slow, and the weight gain of the pups in the HR group was not significantly different from that of the pups in the air groups on PN7 (Fig. 1B, C).

### Differences in apoptosis in brain tissue among groups

**Fig. 2:**
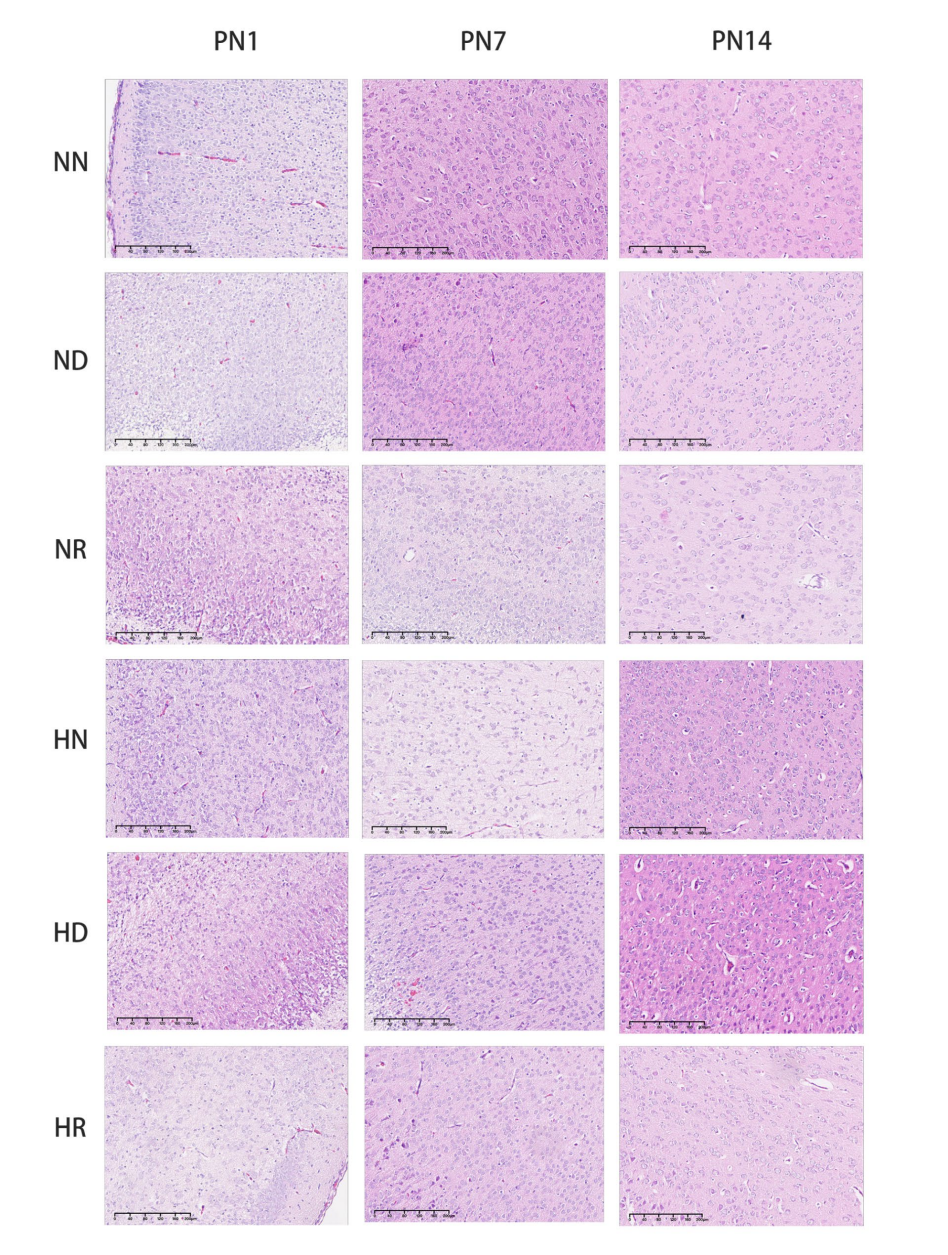
HE staining in NN, ND, NR, HN, HD, and HR groups brain tissues of SD pups on PN1, PN7, and PN14. NN, the nonhyperoxia group; ND, the nonhyperoxia with dimethyl sulfoxide group; NR, the nonhyperoxia with Res group; HN, the hyperoxia group; HD, the hyperoxia with dimethyl sulfoxide group; HR, the hyperoxia with Res group; PN1, postnatal day 1; PN7, postnatal day 7; PN14, postnatal day 14; Res, resveratrol.

**Fig. 3:**
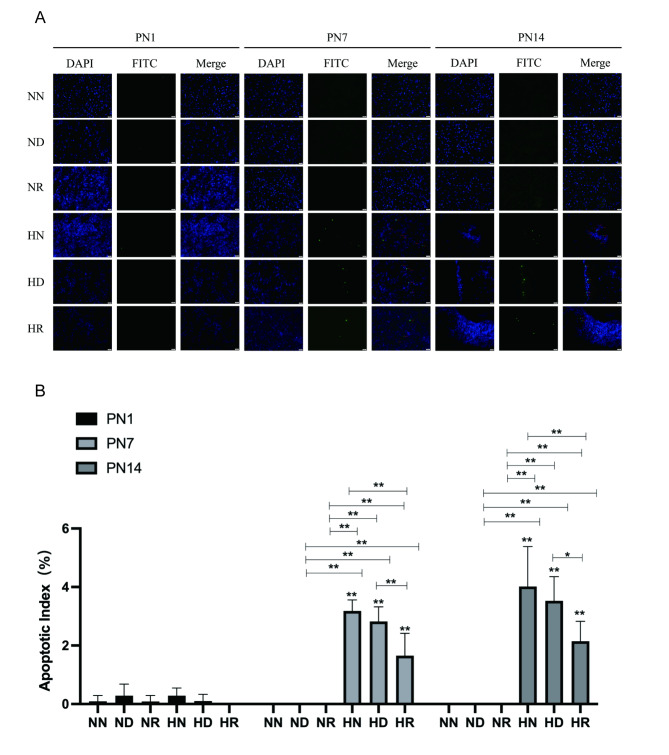
(**A**) TUNEL results in NN, ND, NR, HN, HD, and HR groups brain tissues of SD pups on PN1, PN7, and PN14. (**B**) Apoptosis index in NN, ND, NR, HN, HD, and HR groups on PN1, PN7, and PN14. *p < 0.05, **p < 0.001. NN, the nonhyperoxia group; ND, the nonhyperoxia with dimethyl sulfoxide group; NR, the nonhyperoxia with Res group; HN, the hyperoxia group; HD, the hyperoxia with dimethyl sulfoxide group; HR, the hyperoxia with Res group; PN1, postnatal day 1; PN7, postnatal day 7; PN14, postnatal day 14. DAPI, 4,6-diamino-2-phenyl indole. FITC, fluorescein isothiocyanate; Res, resveratrol.

HE staining and TUNEL staining were used to record the apoptosis of brain tissue from pathological and immunohistochemical aspects, respectively. According to the results of brain HE staining, the morphology of cells at the grey-white matter junction of pups was observed. On the first day after birth, different degrees of glial cell oedema and neuronal apoptosis were found in the brain sections of young pups in each group, which were manifested by the clear cytoplasm of some cells and nuclear pyknosis and nuclear fragmentation in some cells. In the brain slices of pups on PN7 and PN14, the brain tissue of pups in the air environment was different from that of pups in a hyperoxic environment. In the pups from the air environment, the neuronal cells showed normal morphology with large and round nuclei. In the HN group and the HD group, the cell bodies and nuclei of neurons were reduced, and the nuclei were dark ebony and pyknotic. The HR group had less damage (Fig. 2). According to the TUNEL staining results and apoptosis index, apoptotic cells were occasionally found in the brain tissue of pups on PN1, but there was no significant difference between the groups. On PN7, the HN and HD brains exhibited obvious apoptosis signals, and the apoptosis index of the pups in these two groups was higher than that of the pups in the NN, ND, NR, and HR groups, and the difference was statistically significant *(p < 0.05)*. On PN14, the pups in the HN and HD groups had more apoptotic signals, and the apoptosis index was significantly higher than that of the pups in the other four groups *(p < 0.05)* (Fig. 3A, B).

### mRNA levels of PGC-1α, Sirt1, Nrf1, Nrf2, TFAM, and ND1 and the ND4/ND1 ratio were different among the groups

**Fig. 4:**
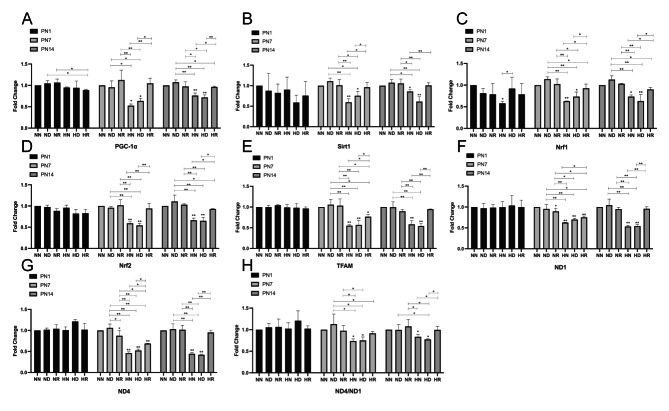
(**A**) The fold change of mRNA expression of PGC-1α in NN, ND, NR, HN, HD, and HR groups on PN1, PN7, and PN14. (**B**) The fold change of mRNA expression of Sirt1 in NN, ND, NR, HN, HD, and HR groups on PN1, PN7, and PN14. (**C**) The fold change of mRNA expression of Nrf1 in NN, ND, NR, HN, HD, and HR groups on PN1, PN7, and PN14. (**D**) The fold change of mRNA expression of Nrf2 in NN, ND, NR, HN, HD, and HR groups on PN1, PN7, and PN14. (**E**) The fold change of mRNA expression of TFAM in NN, ND, NR, HN, HD, and HR groups on PN1, PN7, and PN14. (**F**) The fold change of mRNA expression of ND1 in NN, ND, NR, HN, HD, and HR groups on PN1, PN7, and PN14. (**G**) The fold change of mRNA expression of ND4 in NN, ND, NR, HN, HD, and HR groups on PN1, PN7, and PN14. (**H**) The fold change of mRNA expression of ND4/ND1 in NN, ND, NR, HN, HD, and HR groups on PN1, PN7, and PN14. *p < 0.05, **p < 0.001. NN, the nonhyperoxia group; ND, the nonhyperoxia with dimethyl sulfoxide group; NR, the nonhyperoxia with Res group; HN, the hyperoxia group; HD, the hyperoxia with dimethyl sulfoxide group; HR, the hyperoxia with Res group; PN1, postnatal day 1; PN7, postnatal day 7; PN14, postnatal day 14; PGC-1α, peroxisome proliferator-activated receptor gamma coactivator-1α; Sirt1, silencing information regulator 2-related enzyme 1; Nrf1, nuclear respiratory factor 1; Nrf2, nuclear respiratory factor2; TFAM, mitochondrial transcription factor A; ND1, NADH dehydrogenase 1; ND4, NADH dehydrogenase 4; Res, resveratrol.

Q-PCR showed that there was basically no difference in the mRNA expression of the target genes among the groups on PN1, and occasionally the mRNA expression of PGC-1α and Nrf1 showed irregular statistical significance. On PN7 and PN14, the mRNA expression levels of PGC-1α, Sirt1, Nrf1, Nrf2, TFAM, ND1, and ND4 in the HN and HD groups were lower than those in the NN, ND, NR, and HR groups *(p < 0.05)*. In mitochondrial DNA (mtDNA), the expression of the ND1 gene was positively correlated with that of mtDNA. The expression level of ND4/ND1 was negatively correlated with the degree of mtDNA damage. In conclusion, on PN7 and PN14, the expression of mtDNA in the HN and HD groups was lower and the degree of damage was higher than those in the NN, ND, NR, and HR groups (Fig. 4A-H).

### Protein expression levels of PGC-1α, Sirt1, Nrf1, Nrf2 and TFAM were different among the groups

**Fig. 5:**
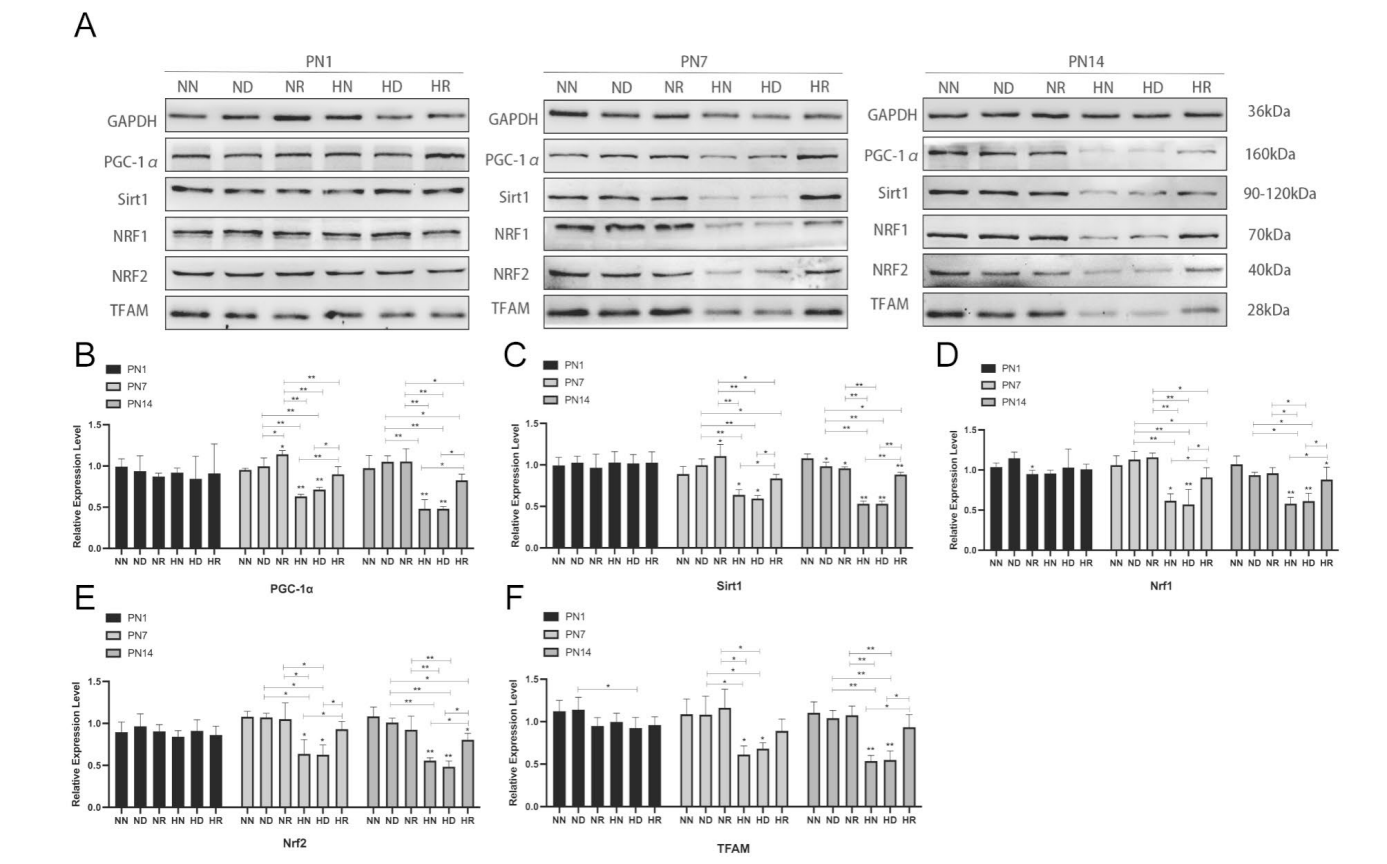
(**A**) The protein blot bands from each group of pups on PN1, PN7, and PN14. All imprints were from the same gel and the PVDF membrane was cut and regenerated according to the molecular weight of the target protein. (**B**) The relative expression of PGC-1α in NN, ND, NR, HN, HD, and HR groups on PN1, PN7, and PN14. (**C**) The relative expression of Sirt1 in NN, ND, NR, HN, HD, and HR groups on PN1, PN7, and PN14. (**D**) The relative expression of Nrf1 in NN, ND, NR, HN, HD, and HR groups on PN1, PN7, and PN14. (**E**) The relative expression of Nrf2 in NN, ND, NR, HN, HD, and HR groups on PN1, PN7, and PN14. (**F**) The relative expression of TFAM in NN, ND, NR, HN, HD, and HR groups at PN1, PN7, and PN14. *p < 0.05, **p < 0.001. NN, the nonhyperoxia group; ND, the nonhyperoxia with dimethyl sulfoxide group; NR, the nonhyperoxia with Res group; HN, the hyperoxia group; HD, the hyperoxia with dimethyl sulfoxide group; HR, the hyperoxia with Res group; PN1, postnatal day 1; PN7, postnatal day 7; PN14, postnatal day 14; PGC-1α, peroxisome proliferator-activated receptor gamma coactivator-1α; Sirt1, silencing information regulator 2-related enzyme 1; Nrf1, nuclear respiratory factor 1; Nrf2, nuclear respiratory factor2; TFAM, mitochondrial transcription factor A; GAPDH, glyceraldehyde-3-phosphate dehydrogenase; Res, resveratrol.

On PN7 and PN14, the expression levels of PGC-1α, Sirt1, Nrf1, Nrf2, and TFAM proteins in the HN and HD groups were downregulated compared with those in the air environment *(p < 0.05)*. There was no difference between the target protein in the HR group and the three groups in the air environment, and the expression of the target protein in the HR group was upregulated compared with that in the HN and HD groups *(p < 0.05)*. There was no difference in protein expression among the NN, ND, and NR groups (Fig. 5A-F).

## Discussion

Compared with neonatal brain tissue, neonatal pups were almost equivalent to 24-week-old human newborns at birth, and pups on the seventh day after birth were almost equivalent to 40 weeks in humans. This period is an important period of myelin formation, dendritic development, synaptic formation, and synaptic modification. [32] Because of the similarity of brain development, most neonatal neurological disease models are studied in rodents. [33] The method of constructing the hyperoxia model used in this experiment has also been verified in many studies. [28, 29] In our study, pathological staining and TUNEL staining were used to verify the apoptosis of brain tissue, and the expression of various factors in the PGC-1α/Nrf/TFAM signalling pathway were observed by q-PCR and western blot at the mRNA and protein levels, thus showing how Res reduces hyperoxia-induced brain injury. According to the research results and previous literature, it can be inferred that ROS caused by hyperoxia can destroy the structure and function of nerve cell mitochondria, leading to a state of oxidative stress in brain tissue and causing cell apoptosis. When the function of mitochondria is damaged, they are unable to carry out self-homeostasis repair, including the inability to complete normal mitochondrial biogenesis, which further aggravates cell death.

**Fig. 6:**
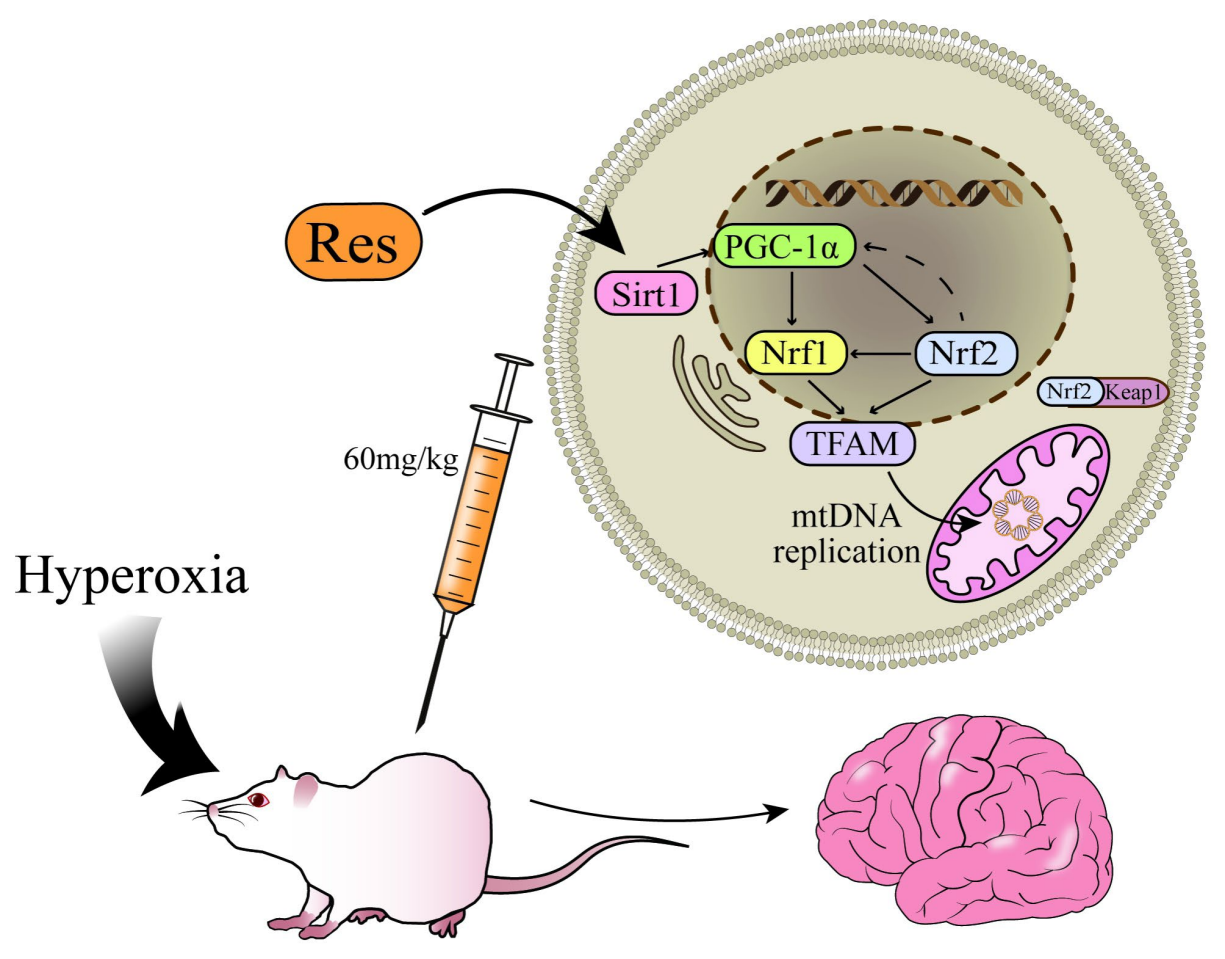
Illustration of how Res reduces hyperoxic brain injury through the PGC-1α/Nrf/TFAM/mtDNA signalling pathway. Res: resveratrol; Sirt1, silencing information regulator 2-related enzyme 1; PGC-1α, peroxisome proliferator-activated receptor gamma coactivator-1α; Nrf1, nuclear respiratory factor 1; Nrf2, nuclear respiratory factor 2; Keap1, Kelch-like ECH associated protein 1; TFAM, mitochondrial transcription factor A; mtDNA, mitochondrial DNA.

On the other hand, Res increases the levels of cyclic adenosine monophosphate (cAMP) and nicotinamide adenine dinucleotide (NAD+) by competitively inhibiting the degradation of phosphodiesterase by cAMP, [34] which is dependent on fluorescence-modified substrate and the N-segment domain of Sirt1 to stimulate Sirt1 upregulation. [35] Sirt1 then deacetylates lysine residues to activate PGC-1α. [20] PGC-1α contains a transcriptional activation domain at the amino-terminus, which is rich in leucine-rich LXXLL sequences, and an RNA binding sequence and host cytokine-1 (HCF) binding domain at the carboxyl-terminus. It can mediate the interaction of transcription factors, including Nrf1, Nrf2, oestrogen-related receptor (ERR), and peroxisome proliferator-activated receptor γ (PPARγ), to enhance mitochondrial biogenesis and oxidation. [36] Nrf1, an endoplasmic reticulum anchor membrane protein, can be activated and phosphorylated upstream by PGC-1α. Phosphorylated Nrf1 undergoes nuclear translocation, binds to the TFAM promoter, and stimulates TFAM transcription. [37] In addition, Nrf1 can also be activated directly by ROS and Nrf2. Recent studies have shown that Nrf1 is a “quality control” factor in the mitochondrial stress response (UPRmt) mechanism. [38] Nrf2 also has an upregulation effect on TFAM after receiving upstream PGC-1α stimulation. In addition, under normal conditions, Nrf2 binds to the inhibitor Kelch-like ECH-associated protein 1 (Keap1) and exists in the cytoplasm in an inactive state. After upstream stimulation, Nrf2 is decoupled from Keap1 and enters the nucleus to stimulate the antioxidant response element (ARE). The PGC-1α promoter contains AREs. The Nrf2/ARE pathway may participate in mitochondrial biogenesis by forming a feedback loop with PGC-1α. [39] TFAM is a nuclear coding protein of 25 kDa whose C-terminal end plays an important role in mtDNA replication. Under the coordination of mitochondrial RNA polymerase (POLRMT) and mitochondrial transcription factor B (TFBM), TFAM can enhance the transcription initiation of the mtDNA light chain promoter (LSP) and carry out D-loop replication. This improves mtDNA replication. [40, 41] In conclusion, Res can promote the expression of the PGC-1α/Nrf/TFAM signalling pathway by upregulating the expression of Sirt1, increasing the amount of mtDNA replication and carrying out mitochondrial biogenesis in cells, thus protecting brain tissue from oxidative stress damage (Fig. 6).

The protection of brain tissue by mitochondrial biogenesis is divided into several aspects, and the increase in mitochondria can better provide energy for a series of physiological activities of nerve cells. Mitochondria provide 90% of the energy needed by cells through oxidative phosphorylation, [42] while the brain consumes approximately 20% of its energy at rest. [43] Thus, the normal function of the developing brain depends heavily on the synthesis of adenosine triphosphate (ATP), which can maintain neuronal excitability and synaptic function. [44] For example, chromatin remodelling factors that regulate neurogenesis in the cerebral cortex are highly ATP dependent. [45] In addition, the brain has a certain correction mechanism, that is, neural plasticity and synaptic plasticity. This means that after being damaged, the brain can mitigate the adverse effects of injury through a series of behaviours, such as neurogenesis, synaptic formation, and reorganization can regulate this plasticity of cells. [46] Hyperoxia has been shown to interfere with the regulation of genes related to synaptic plasticity in rodents and destroy neuroplasticity and synaptic plasticity in the brain. [47] Mitochondria maintain the normal operation of plasticity mainly through three aspects, including a large ATP energy supply, buffering calcium ions in synapses to reduce excessive excitotoxicity, [48, 49] and producing physiological ROS to regulate synaptic transmission. [50] Therefore, it is speculated that after mitochondrial biogenesis in brain tissue, mtDNA replication and the number of mitochondria increase, which improves the insufficient energy supply of nerve cells and alleviates brain injury and its long-term effects by improving synaptic plasticity and neuroplasticity efficiency.

However, our experiment failed to observe long-term behavioural changes in rats, and it is not clear whether Res can improve the neuroplasticity of neonatal rat brain tissue through mitochondrial biogenesis. In addition to activating TFAM, PGC-1α can also participate in neuroprotection by activating other downstream effectors, such as uncoupling protein-1 (UCP-1) and b-cell lymphoma-2 (Bcl-2), to antagonize oxidative stress, alleviate mitochondrial dysfunction, relieve neuroinflammation, and reduce autophagy and apoptosis. [51] Here, we only discuss how TFAM protects the brain from hyperoxia-induced injury through mitochondrial biogenesis.

In addition, our group observed hyperoxia-induced injury of the lungs, brain, and kidneys in the same hyperoxic newborn SD pups model. After administering the same dose of Res, the damage to the three organs decreased to varying degrees. It is speculated that the protective effect of Res on hyperoxic organ injury is systemic. [19] Unfortunately, we have not been able to conduct a horizontal study of multiple organs in the same mouse at the same time to verify this conjecture, and we are unable to further study the sequence or causality of organ damage.

It is important to study the difference in injuries among organs after hyperoxia in the same experimental model. BPD and neurodysplasia are closely related to each other. At present, there are many hypotheses about the relationship between them, including the release of extracellular vesicles (Evs) loaded with Gasdermin D protein by pulmonary epithelial cells, which causes pyroptosis, [52] or the cessation of insulin-like growth factor-1 (IGF-1) supplied by the placenta. [53] Observing the injury of various tissues and organs at the same time may be helpful to determine the relationship between the two.

However, in the HE staining and TUNEL results of the hyperoxia-induced brain injury model, we found that there were different degrees of apoptosis in the brain tissue of neonatal pups in the PN1 group but not in the lung and kidney. Generally, the duration of hyperoxia in lung injury in SD pups is 10–14 days, the duration of the brain injury model is 6–48 h, and the oxygen concentration used in the BPD experimental model is 85%, while that of the premature encephalopathy model is 80%. [54] Rantakari et al. also demonstrated that some areas of the brain tissue of very premature infants are sensitive to hyperoxia by observing secondary cortical somatosensory processing in magnetoencephalography (MEG-SII). [55] Among all hyperoxic organ injuries, brain tissue is the most sensitive to oxidative stress and has selective vulnerability. For example, neurons in the hippocampal CA1 region are more vulnerable to oxidative stress. [56] Hyperoxia-induced damage to the hippocampus of mice leads to learning cognitive impairment. [4].

Among 53 million children with developmental disabilities under the age of 5, nearly 80% of disabilities are associated with perinatal brain injury. [57] Oxidative stress caused by hyperoxia is an important cause of perinatal brain injury. Hyperoxia can damage neurons and microvessels in all regions of the brain, [58] disrupting normal neurological function and development. The effect of hyperoxia on neonatal neurodevelopment can be maintained in childhood and even in adulthood. Hyperoxia can damage memory function in newborn mice. [59] Scheuer et al. speculated that oxidative stress damage after preterm delivery may also lead to long-term autism, attention deficit disorder, and other conditions. [60] Consequently, the study of the protective mechanism of Res on hyperoxic brain injury can provide a potential therapeutic target for perinatal brain injury and is of great significance to alleviate the familial and socioeconomic burden.

## Conclusion

Neonatal pups with hyperoxia-induced injury showed gait stumbling, lethargy, weight loss, and nerve cell injury on PN7 and PN14, and the levels of Sirt1, PGC-1α, Nrf1, Nrf2, and TFAM decreased. Immediately after hyperoxia, Res reduced injury; increased the expression of Sirt1, PGC-1α, Nrf1, Nrf2, and TFAM; upregulated the expression of mtDNA; reduced mitochondrial damage; and improved the growth of neonatal rats.

In summary, we once again verified that our hyperoxia model can damage the brain tissue of neonatal pups. At the same time, Res can stimulate mitochondrial biogenesis through the PGC-1α/Nrf/TFAM/mtDNA signalling pathway, increase the number of mitochondria in nerve cells, and thus have a protective effect on hyperoxia-induced brain injury in neonatal pups.

## Electronic supplementary material

Below is the link to the electronic supplementary material.

Supplementary Material 1

## Data Availability

The datasets generated and analysed during the current study are not publicly available due to avoid data leakage, but are available from the corresponding author on reasonable request.

## List of Abbreviations

ARE: Antioxidant response element
ATP: Adenosine triphosphate
Bcl-2: B-cell lymphoma-2
BDNF: Brain-derived neurotrophic factor
BPD: Pulmonary dysplasia
cAMP: Cyclic adenosine monophosphate
DMSO: Dimethyl sulfoxide
ERR: Estrogen related receptor
Ev: Extracellular vesicle
HCF: Host cytokine − 1
HD: The hyperoxia with dimethyl sulfoxide group
HN: The hyperoxia group
HR: The hyperoxia with Resveratrol group
IGF-1: Insulin-like growth factor-1
IRS: Insulin receptor substrate
Keap1: Kelch-like ECH-associated protein1
LSP: Light chain promoter
MEG-SII: Magnetoencephalography
mtDNA: Mitochondrial DNA
NAD+: Nicotinamide adenine dinucleotide
ND: The non-hyperoxia with dimethyl sulfoxide group
NLRP3: Nod-like receptor pyrin domain-containing-3
NN: The non-hyperoxia group
NR: The non-hyperoxia with Resveratrol group
Nrf1: Nuclear respiratory factor 1
Nrf2: Nuclear respiratory factor2
OAA: Oxalyl acetate
PGC-1α: Peroxisome proliferator-activated receptor gamma coactivator-1α
PN1: Postnatal day 1
PN14: Postnatal day 14
PN7: Postnatal day 7
POLRMT: Mitochondrial RNA polymerase
PPARγ: Peroxisome proliferator-activated receptor γ
q-PCR: Real-time quantitative polymerase chain reaction
Res: Resveratrol
ROS: Reactive oxygen species
SD rat: Sprague-Dawley rat
Sirt1: Silencing information regulator 2-related enzyme 1
TFAM: Mitochondrial transcription factor A
TFBM: Mitochondrial transcription factor B
TH: Thyroid hormone
TUNEL: Terminal-deoxynucleotidyl transferase mediated nick end labeling
UCP-1: Uncoupling protein-1
UPRmt: Mitochondrial stress response

## References

[1] Angusamy S, Mansour T, Abdulmageed M, Han R, Schutte BC, LaPres J (2018). Altered thymocyte and T cell development in neonatal mice with hyperoxia-induced lung injury. J Perinat Med.

[2] Micili SC, Engür D, Genc S, Ercan I, Soy S, Baysal B (2020). Oxygen exposure in early life activates NLRP3 inflammasome in mouse brain. Neurosci Lett.

[3] Schmitz T, Ritter J, Mueller S, Felderhoff-Mueser U, Chew LJ, Gallo V (2011). Cellular changes underlying hyperoxia-induced delay of white matter development. J Neurosci.

[4] Abbah J, Vacher CM, Goldstein EZ, Li Z, Kundu S, Talbot B (2022). Oxidative Stress-Induced damage to the developing Hippocampus is mediated by GSK3β. J Neurosci.

[5] Lithopoulos MA, Toussay X, Zhong S, Xu L, Mustafa SB, Ouellette J et al. Neonatal hyperoxia in mice triggers long-term cognitive deficits via impairments in cerebrovascular function and neurogenesis. J Clin Invest. 2022;132(22). 10.1172/JCI146095 PMC9663164 36136598

[6] Bouchez C, Devin A. Mitochondrial Biogenesis and Mitochondrial Reactive Oxygen Species (ROS): A Complex Relationship Regulated by the cAMP/PKA Signaling Pathway. Cells. 2019;8(4). 10.3390/cells8040287 PMC6523352 30934711

[7] Brugniaux JV, Coombs GB, Barak OF, Dujic Z, Sekhon MS, Ainslie PN (2018). Highs and lows of hyperoxia: physiological, performance, and clinical aspects. Am J Physiol Regul Integr Comp Physiol.

[8] Brand MD (2010). The sites and topology of mitochondrial superoxide production. Exp Gerontol.

[9] Aquilano K, Baldelli S, Pagliei B, Cannata SM, Rotilio G, Ciriolo MR (2013). p53 orchestrates the PGC-1α-mediated antioxidant response upon mild redox and metabolic imbalance. Antioxid Redox Signal.

[10] Pérez S, Rius-Pérez S, Finamor I, Martí-Andrés P, Prieto I, García R (2019). Obesity causes PGC-1α deficiency in the pancreas leading to marked IL-6 upregulation via NF-κB in acute pancreatitis. J Pathol.

[11] Wu J, Li J, Feng B, Bi Z, Zhu G, Zhang Y (2022). Activation of AMPK-PGC-1α pathway ameliorates peritoneal dialysis related peritoneal fibrosis in mice by enhancing mitochondrial biogenesis. Ren Fail.

[12] Sun J, Leng P, Li X, Guo Q, Zhao J, Liang Y (2022). Salvianolic acid a promotes mitochondrial biogenesis and mitochondrial function in 3T3-L1 adipocytes through regulation of the AMPK-PGC1α signalling pathway. Adipocyte.

[13] Alattar A, Alshaman R, Al-Gayyar MMH (2022). Therapeutic effects of sulforaphane in ulcerative colitis: effect on antioxidant activity, mitochondrial biogenesis and DNA polymerization. Redox Rep.

[14] Tomé-Carneiro J, Carmen Crespo M, Burgos-Ramos E, Tomas-Zapico C, García-Serrano A, Castro-Gómez P (2018). Buttermilk and Krill Oil Phospholipids improve hippocampal insulin resistance and synaptic signaling in aged rats. Mol Neurobiol.

[15] Liu Z, Patil IY, Jiang T, Sancheti H, Walsh JP, Stiles BL (2015). High-fat diet induces hepatic insulin resistance and impairment of synaptic plasticity. PLoS ONE.

[16] Wilkins HM, Harris JL, Carl SM, Lu EL, Eva Selfridge J (2014). Oxaloacetate activates brain mitochondrial biogenesis, enhances the insulin pathway, reduces inflammation and stimulates neurogenesis. Hum Mol Genet.

[17] Weitzel JM, Iwen KA (2011). Coordination of mitochondrial biogenesis by thyroid hormone. Mol Cell Endocrinol.

[18] Teng YC, Tai YI, Huang HJ, Lin AM (2015). Melatonin ameliorates Arsenite-Induced neurotoxicity: involvement of Autophagy and Mitochondria. Mol Neurobiol.

[19] Yang K, Yang M, Shen Y, Kang L, Zhu X, Dong W et al. Resveratrol Attenuates Hyperoxia Lung Injury in Neonatal Rats by Activating SIRT1/PGC-1α Signaling Pathway. Am J Perinatol. 2022. 10.1055/a-1787-3396 35240708

[20] Quan Y, Xin Y, Tian G, Zhou J, Liu X (2020). Mitochondrial ROS-Modulated mtDNA: a potential target for Cardiac Aging. Oxid Med Cell Longev.

[21] Shaito A, Posadino AM, Younes N, Hasan H, Halabi S, Alhababi D et al. Potential Adverse Effects of Resveratrol: A Literature Review. Int J Mol Sci. 2020;21(6). 10.3390/ijms21062084 PMC7139620 32197410

[22] Qi J, Fu LY, Liu KL, Li RJ, Qiao JA, Yu XJ et al. Resveratrol in the Hypothalamic Paraventricular Nucleus Attenuates Hypertension by Regulation of ROS and Neurotransmitters. Nutrients. 2022;14(19). 10.3390/nu14194177 PMC9573276 36235829

[23] Wei S, Liu TT, Hu WP, Qiu CY (2022). Resveratrol inhibits the activity of acid-sensing ion channels in male rat dorsal root ganglion neurons. J Neurosci Res.

[24] Sümer Coşkun A, Bedel HA, Munzuroğlu M, Derin N, Usta C (2022). Does Resveratrol prevent sevoflurane toxicity in newborn rats?. J Med Food.

[25] McCreary MR, Schnell PM, Rhoda DA (2022). Randomized double-blind placebo-controlled proof-of-concept trial of resveratrol for outpatient treatment of mild coronavirus disease (COVID-19). Sci Rep.

[26] Mahjabeen W, Khan DA, Mirza SA (2022). Role of resveratrol supplementation in regulation of glucose hemostasis, inflammation and oxidative stress in patients with diabetes mellitus type 2: a randomized, placebo-controlled trial. Complement Ther Med.

[27] Faghihzadeh F, Adibi P, Hekmatdoost A (2015). The effects of resveratrol supplementation on cardiovascular risk factors in patients with non-alcoholic fatty liver disease: a randomised, double-blind, placebo-controlled study. Br J Nutr.

[28] Kang L, Dong W, Li X, Ruan Y, Zhang R (2021). Resveratrol relieves Hyperoxia-Induced Brain Injury in neonatal rats by activating Sirt1. Am J Perinatol.

[29] Kang L, Dong W, Ruan Y, Zhang R, Wang X (2019). The molecular mechanism of Sirt1 Signaling Pathway in Brain Injury of Newborn rats exposed to Hyperoxia. Biol Pharm Bull.

[30] Ruan Y, Dong W, Kang L, Lei X, Zhang R, Wang F (2020). The changes of Twist1 pathway in pulmonary microvascular permeability in a newborn rat model of Hyperoxia-Induced Acute Lung Injury. Front Pead.

[31] Zhu X, Lei X, Wang J, Dong W. Protective effects of resveratrol on hyperoxia-induced lung injury in neonatal rats by alleviating apoptosis and ROS production. The journal of maternal-fetal & neonatal medicine: the official journal of the European Association of Perinatal Medicine, the Federation of Asia and Oceania Perinatal Societies, the International Society of Perinatal Obstet. 2020;33(24):4150-58. 10.1080/14767058.2019.1597846 30890012

[32] Chini M, Hanganu-Opatz IL (2021). Prefrontal Cortex Development in Health and Disease: Lessons from rodents and humans. Trends Neurosci.

[33] Semple BD, Blomgren K, Gimlin K, Ferriero DM, Noble-Haeusslein LJ (2013). Brain development in rodents and humans: identifying benchmarks of maturation and vulnerability to injury across species. Prog Neurobiol.

[34] Yu L, Li S, Tang X, Li Z, Zhang J, Xue X (2017). Diallyl trisulfide ameliorates myocardial ischemia-reperfusion injury by reducing oxidative stress and endoplasmic reticulum stress-mediated apoptosis in type 1 diabetic rats: role of SIRT1 activation. Apoptosis.

[35] Cao D, Wang M, Qiu X, Liu D, Jiang H, Yang N (2015). Structural basis for allosteric, substrate-dependent stimulation of SIRT1 activity by resveratrol. Genes Dev.

[36] Scarpulla RC, Vega RB, Kelly DP (2012). Transcriptional integration of mitochondrial biogenesis. Trends Endocrinol Metab.

[37] Piantadosi CA, Suliman HB (2006). Mitochondrial transcription factor A induction by redox activation of nuclear respiratory factor 1. J Biol Chem.

[38] Hu S, Feng J, Wang M, Wufuer R, Liu K, Zhang Z (2022). Nrf1 is an indispensable redox-determining factor for mitochondrial homeostasis by integrating multi-hierarchical regulatory networks. Redox Biol.

[39] Gureev AP, Shaforostova EA, Popov VN (2019). Regulation of mitochondrial Biogenesis as a way for active longevity: Interaction between the Nrf2 and PGC-1α signaling pathways. Front Genet.

[40] Kanki T, Ohgaki K, Gaspari M, Gustafsson CM, Fukuoh A, Sasaki N (2004). Architectural role of mitochondrial transcription factor A in maintenance of human mitochondrial DNA. Mol Cell Biol.

[41] Kukat C, Larsson NG (2013). mtDNA makes a U-turn for the mitochondrial nucleoid. Trends Cell Biol.

[42] Mahmud S, Biswas S, Afrose S, Mita MA, Hasan MR, Shimu MSS (2022). Use of Next-Generation sequencing for identifying mitochondrial Disorders. Curr Issues Mol Biol.

[43] Camandola S, Mattson MP (2017). Brain metabolism in health, aging, and neurodegeneration. Embo j.

[44] Rangaraju V, Calloway N, Ryan TA (2014). Activity-driven local ATP synthesis is required for synaptic function. Cell.

[45] Sokpor G, Castro-Hernandez R, Rosenbusch J, Staiger JF, Tuoc T (2018). ATP-Dependent chromatin remodeling during cortical neurogenesis. Front Neurosci.

[46] Lisowski P, Kannan P, Mlody B, Prigione A. Mitochondria and the dynamic control of stem cell homeostasis. EMBO Rep. 2018;19(5). 10.15252/embr.201745432 PMC5934764 29661859

[47] Reich B, Hoeber D, Bendix I, Felderhoff-Mueser U (2016). Hyperoxia and the immature brain. Dev Neurosci.

[48] Todorova V, Blokland A (2017). Mitochondria and synaptic plasticity in the mature and aging nervous system. Curr Neuropharmacol.

[49] Tang J, Oliveros A, Jang MH (2019). Dysfunctional mitochondrial bioenergetics and synaptic degeneration in Alzheimer Disease. Int Neurourol J.

[50] Guo L, Tian J, Du H (2017). Mitochondrial dysfunction and synaptic transmission failure in Alzheimer’s Disease. J Alzheimers Dis.

[51] Lv J, Jiang S, Yang Z, Hu W, Wang Z, Li T (2018). PGC-1α sparks the fire of neuroprotection against neurodegenerative disorders. Ageing Res Rev.

[52] Ali A, Zambrano R, Duncan MR, Chen S, Luo S, Yuan H (2021). Hyperoxia-activated circulating extracellular vesicles induce lung and brain injury in neonatal rats. Sci Rep.

[53] Kramer BW, Niklas V, Abman S. Bronchopulmonary dysplasia and impaired neurodevelopment-what may be the missing link? Am J Perinatol. 2022;11(1):8791. 10.1055/s-0042-1756677 36318942

[54] Obst S, Herz J, Alejandre Alcazar MA, Endesfelder S, Möbius MA, Rüdiger M (2022). Perinatal Hyperoxia and Developmental Consequences on the Lung-Brain Axis. Oxid Med Cell Longev.

[55] Rantakari K, Rinta-Koski OP, Metsäranta M, Hollmén J, Särkkä S, Rahkonen P (2021). Early oxygen levels contribute to brain injury in extremely preterm infants. Pediatr Res.

[56] Wang X, Michaelis EK (2010). Selective neuronal vulnerability to oxidative stress in the brain. Front Aging Neurosci.

[57] Krishnan V, Kumar V, Variane GFT, Carlo WA, Bhutta ZA, Sizonenko S (2021). Need for more evidence in the prevention and management of perinatal asphyxia and neonatal encephalopathy in low and middle-income countries: a call for action. Semin Fetal Neonatal Med.

[58] Sirinyan M, Sennlaub F, Dorfman A, Sapieha P, Gobeil F, Hardy P (2006). Hyperoxic exposure leads to nitrative stress and ensuing microvascular degeneration and diminished brain mass and function in the immature subject. Stroke.

[59] Ramani M, Kumar R, Halloran B, Lal CV, Ambalavanan N, McMahon LL (2018). Supraphysiological levels of oxygen exposure during the neonatal period impairs signaling pathways required for learning and memory. Sci Rep.

[60] Scheuer T, dem Brinke EA, Grosser S, Wolf SA, Mattei D, Sharkovska Y et al. Reduction of cortical parvalbumin-expressing GABAergic interneurons in a rodent hyperoxia model of preterm birth brain injury with deficits in social behavior and cognition. Development. 2021;148(20). 10.1242/dev.198390 34557899

