# Supplementary material for: Protective effect of resveratrol on mitochondrial biogenesis during hyperoxia-induced brain injury in neonatal pups
Source: BMC Neurosci. 2023 Apr 25;24:27. doi: 10.1186/s12868-023-00797-1 (PMC10127954; doi:10.1186/s12868-023-00797-1)

PN1-GAPDH

36kDa

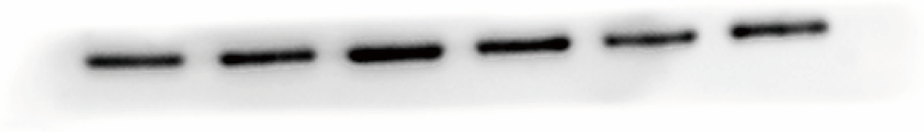

NN ND NR HN HD HR

PN1-PGC1α

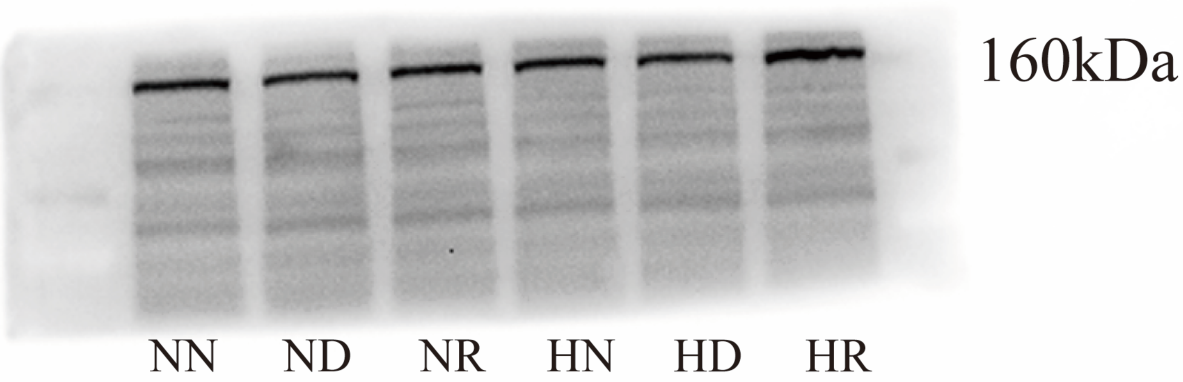

PN1-Sirt1

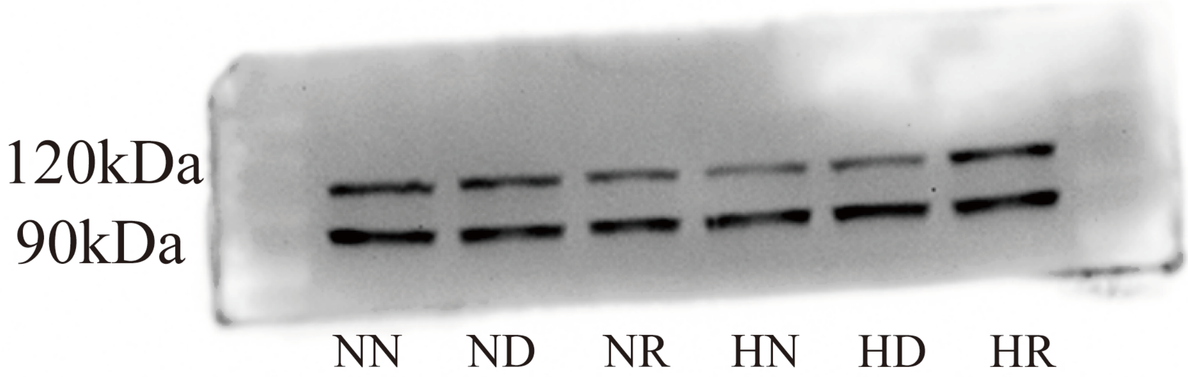

PN1-Nrf1

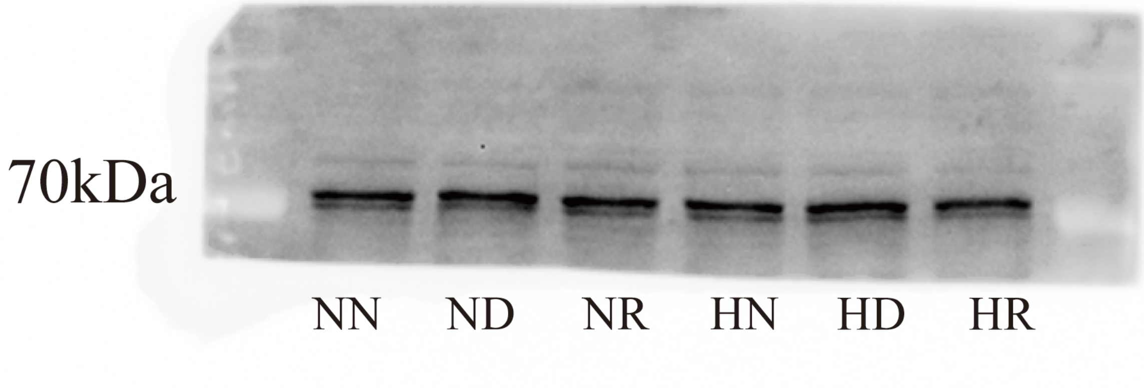

PN1-Nrf2

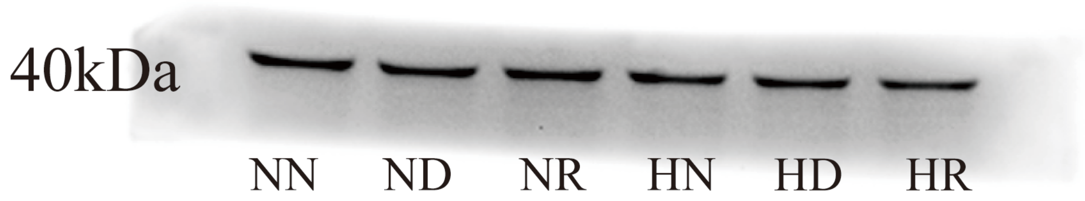

PN1-TFAM

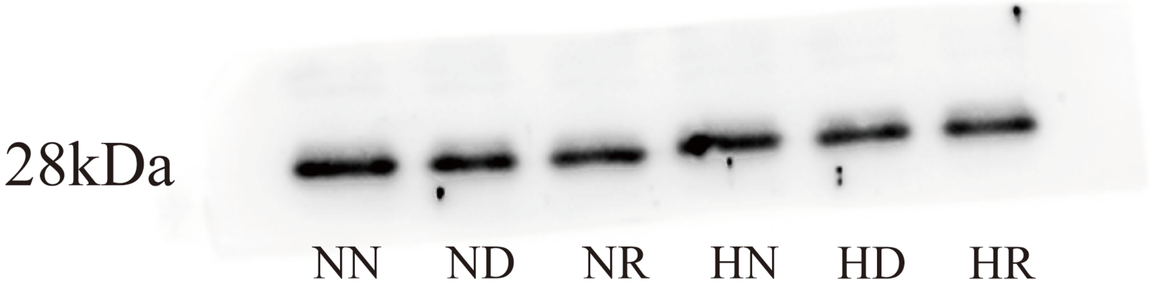

PN7-GAPDH

36kDa

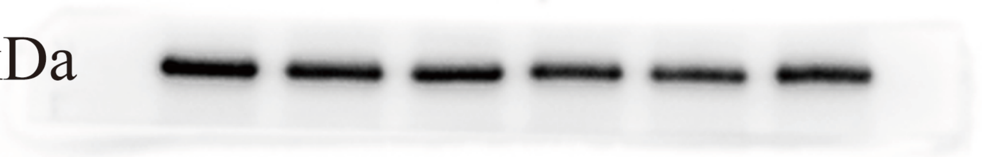

NN ND NR HN HD HR

PN7-PGC1α

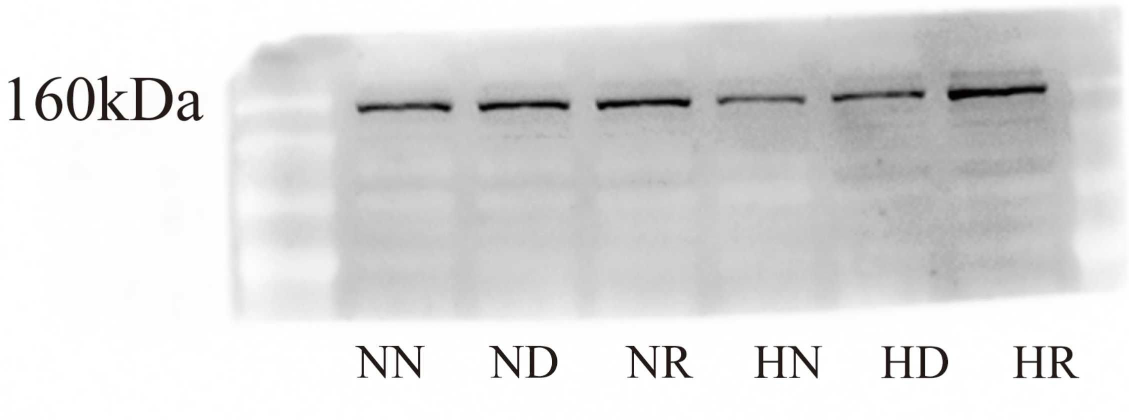

PN7-Sirt1

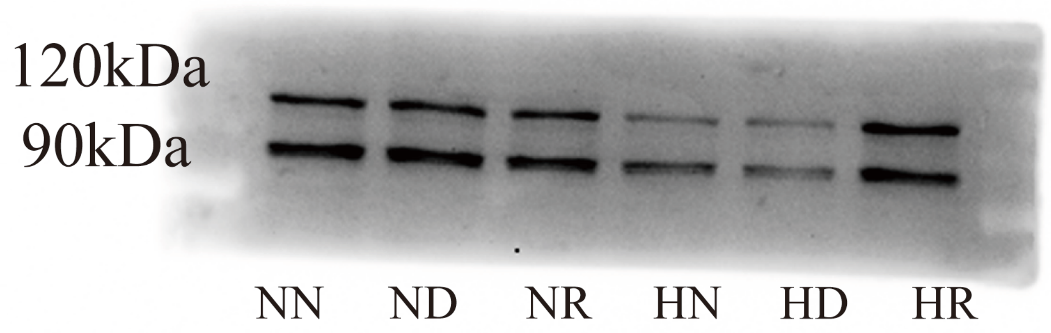

PN7-Nrf1

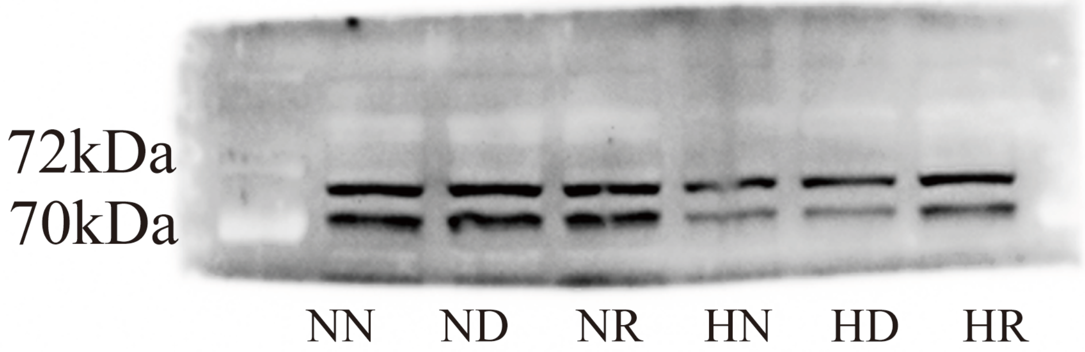

PN7-Nrf2

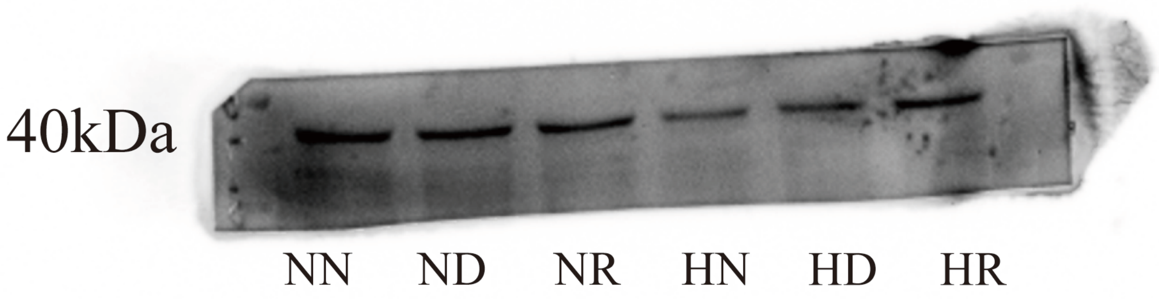

PN7-TFAM

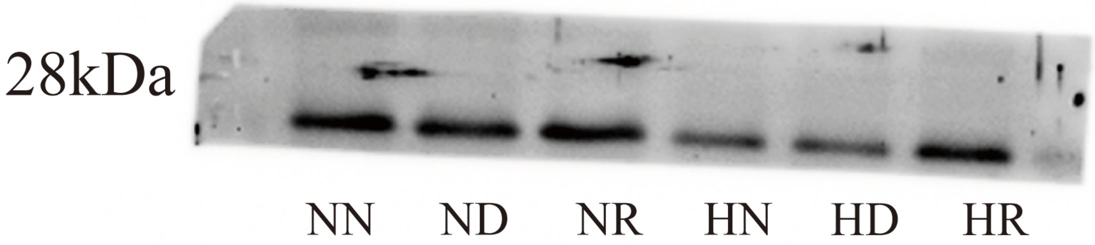

PN14-GAPDH

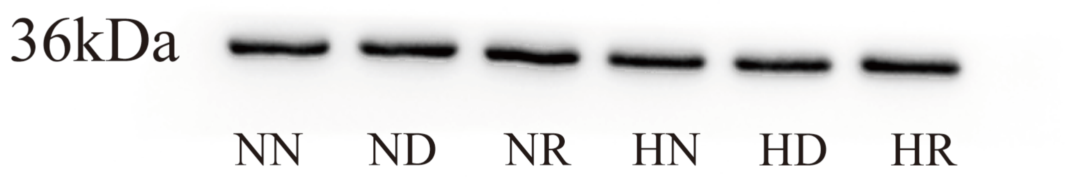

PN14-PGC1α

160kDa

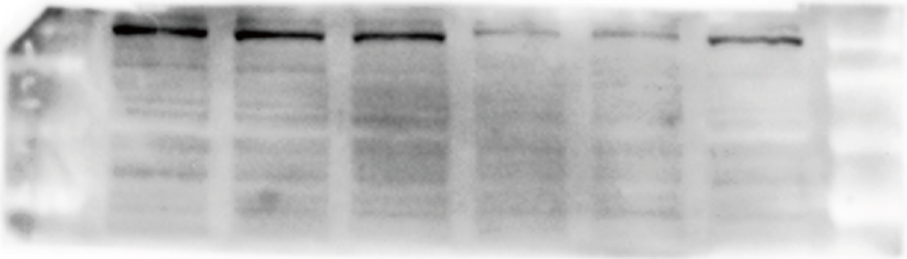

NN ND NR HN HD HR

PN14-Sirt1

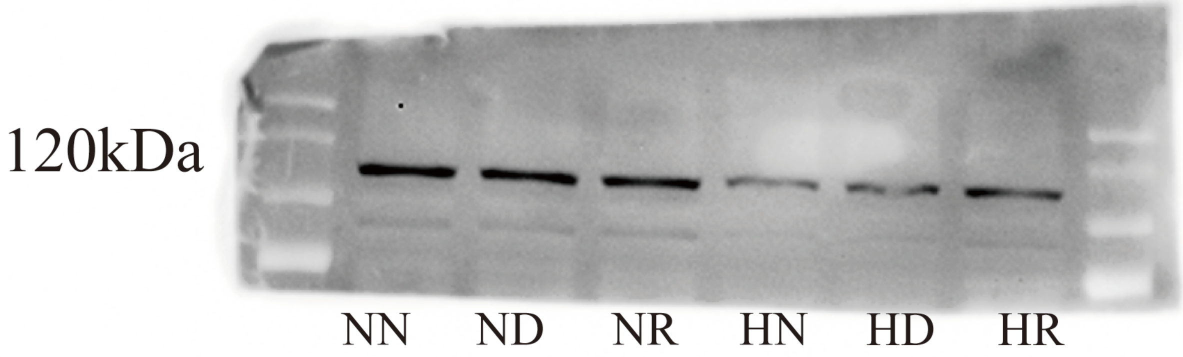

PN14-Nrf1

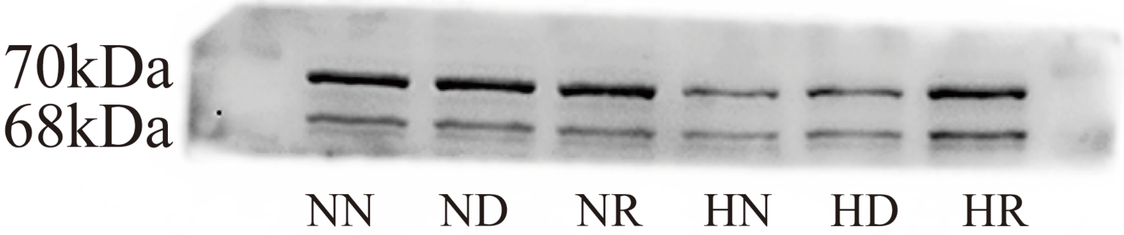

PN14-Nrf2

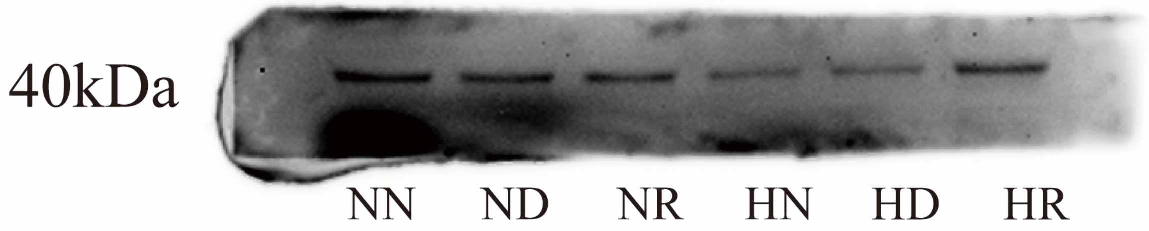

PN14-TFAM

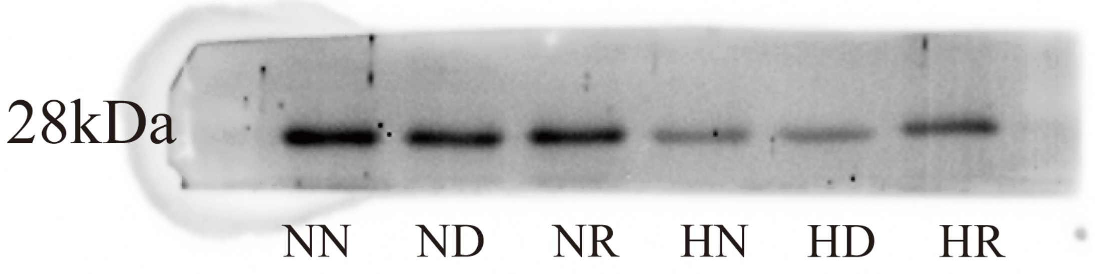

Supplement: Supplementary file 1 — Supplementary Material 1 [file 12868_2023_797_MOESM1_ESM.pdf]
